# Identification and Stability Assessment of Reference Genes in *Helicoverpa armigera* Under Plant Secondary Substance and Insecticide Stresses

**DOI:** 10.3390/biology15020175

**Published:** 2026-01-17

**Authors:** Jie Zhao, Hao-Ran Kan, Xin-Xin Jin, Jiang-Yuan Zhang, Hong-Run Zhou, Xiao-Qiang Han, Jing Ye

**Affiliations:** 1 Key Laboratory of Xinjiang for Oasis Agricultural Pests Management and Plant Protection Resources Utilization, Agricultural College, Shihezi University, Shihezi 832003, China; zhaojie198967@163.com (J.Z.); kanhaoran@stu.shzu.edu.cn (H.-R.K.); jxx@163.com (X.-X.J.); zhangjiangyuanda@163.com (J.-Y.Z.); 2 Laboratory of Forestry Department, College of Urban and Environmental Sciences, Shihezi University, Shihezi 832003, China; zhouhongrun@stu.shzu.edu.cn

**Keywords:** *Helicoverpa armigera*, reference gene, plant secondary substance, insecticide, *GADD45*

## Abstract

The cotton bollworm (*Helicoverpa armigera*, Lepidoptera: Noctuidae) is a globally distributed agricultural pest. When conducting expression analysis of its functional genes, appropriate reference genes should be selected to ensure the reliability of the results. In this study, five algorithms including Delta Ct, GeNorm, Normfinder, BestKeeper, and RefFinder were used to evaluate the expression stability of eleven candidate reference genes under different developmental stages, larval tissues, adult sexes, plant secondary substance stresses, and insecticide treatments in *H. armigera*. The candidate genes included *Actin*, *Tubulin*, *EF-1α*, *RPS3*, *RPS15*, *RPL27*, *RPL32*, *28S*, *GAPDH*, *SOD*, and *TRX*. The reliability of the recommended reference gene combinations was validated using the growth arrest and DNA-damage-inducible gene 45 (*GADD45*). The results showed that normalizing relative expression of the target gene with the combination of the two most stable reference genes is recommended. Specifically, the combination of *RPS3* + *RPL27* is recommended for developmental stage comparisons; *RPL32* + *RPL27* for larval tissues; *RPS3* + *RPL27* for adult sex comparisons; *GAPDH* + *RPL32* under tannic acid stress; *RPL32* + *RPS3* under quercetin stress; *RPS15* + *RPL32* under 2-tridecanone stress; *RPS3* + *RPL32* under ZQ-8 stress; *RPL27* + *TRX* following chlorantraniliprole treatment; and *RPL27* + *RPL32* following indoxacarb treatment. Moreover, larvae exposed to three concentrations of plant secondary substances and to sublethal doses of insecticides exhibited significant upregulation of *GADD45*: after 4 h of exposure to 1% tannic acid, 0.1% and 1% quercetin, 1% 2-tridecanone, and 0.05% ZQ-8; after 15 h of chlorantraniliprole treatment; and after 24 h of indoxacarb treatment. Thus, *GADD45* was overexpressed in response to various plant secondary substances and insecticide treatments, indicating its involvement in the detoxification and metabolism of *H. armigera*. This study proves to be helpful for selecting reference genes in *H. armigera* under plant secondary substance and insecticide stress and lays the foundation for further research utilizing *GADD45* as a molecular target for pest control.

## Simple Summary

The cotton bollworm (*Helicoverpa armigera*), a globally significant agricultural pest, displays notable host adaptability and insecticide resistance. This study utilized five algorithms to assess the expression stability of eleven candidate reference genes in *H. armigera* under stress from four plant secondary substances (tannic acid, quercetin, 2-tridecanone, and ZQ-8) and two insecticides (chlorantraniliprole and indoxacarb). The findings indicated that the optimal combination of reference genes varied across different stress conditions. Additionally, ribosomal protein *RPL32* and *RPL27* exhibited relatively consistent expression levels under plant secondary substance and insecticide treatments, respectively. Additionally, larvae exposed to sublethal doses of plant secondary substances and insecticides showed a significant upregulation of growth arrest and DNA-damage-inducible gene 45 (*GADD45*) expression, indicating its involvement in the detoxification metabolism of *H. armigera*. This research establishes a standardized foundation for using real-time quantitative polymerase chain reaction (qPCR) to explore the molecular regulatory pathways of *H. armigera* in response to plant secondary substance and insecticide stress. It also identifies potential novel target sites for the management of insecticide resistance in this pest.

## 1. Introduction

In molecular biology, real-time quantitative polymerase chain reaction (qPCR) is a highly sensitive, specific, simple, and efficient molecular technique. It is widely employed to quantify gene transcription levels by detecting mRNA abundance [1]. The measurement of mRNA levels can vary in fold changes due to several factors, including RNA quality, pipetting errors, PCR amplification efficiency, and so on [2,3]. Furthermore, nearly all studies analyzing gene expression utilize an internal standard to normalize mRNA levels across different samples, commonly referred to as a reference gene [4]. The expression of genes within an organism can be influenced by developmental or environmental changes. Some genes may exhibit minor variations, while others may show significant fluctuations. Consequently, the selection of reliable reference genes has become a critical step in gene expression studies [5,6,7]. An ideal reference gene should maintain relatively consistent expression levels across various conditions without substantial alterations. The use of an inappropriate reference gene may lead to erroneous conclusions. To fulfill this criterion, reference genes are typically selected from those that regulate basic cellular functions and/or maintain cellular structures [8]. Examples include Actin, Tubulin, ribosomal protein L (RPL), ribosomal protein S (RPS), glyceraldehyde-3-phosphate dehydrogenase (GAPDH), 18S ribosomal RNA (18S), 28S ribosomal RNA (28S), elongation factor 1 alpha (EF1-α), and Cu/Zn superoxide dismutase (SOD), among others [9,10].

The cotton bollworm, *Helicoverpa armigera* (Hübner), is a polyphagous lepidopteran pest. Its larvae feed on the tender leaves, flower buds, or fruits of over 200 plant species from more than 30 families, and it has developed varying degrees of resistance to most commonly used insecticides [11,12]. Since the late 20th century, the affected area and damage severity by cotton bollworm have been escalating, challenging traditional chemical control methods [13,14]. Despite the introduction of Bt cotton, the issue of insecticide resistance in cotton bollworm persists, necessitating immediate attention in modern agriculture [15]. Understanding the functions of key regulatory genes at the molecular level is crucial for the effective control of *H. armigera* [16]. Currently, three articles have been published addressing the screening and validation of conventional reference genes for functional gene studies in *H. armigera*. These studies encompass a range of biological and non-biological treatments, including developmental stages, larval and adult tissues, dsRNA treatment, temperature stress, photoperiod, starvation stress, mechanical injury, nuclear polyhedrosis virus (NPV) infection, and insecticide-induced stress [17,18,19]. The findings displayed that reference genes exhibit significant variability in expression levels across different treatment conditions, and no single reference gene was universally applicable to all experimental contexts. Additionally, while all three articles evaluated reference genes for developmental stages, each recommended different optimal reference genes [17,18,19]. These results underscore the necessity of validating the stability of specific reference genes under different treatments prior to conducting qPCR experiments.

In this study, we selected eleven previously reported reference genes of cotton bollworm as candidates, including *Actin*, *Tubulin*, *EF1-α*, *RPS3*, *RPS15*, *RPL27*, *RPL32*, *28S*, *GAPDH*, *SOD*, and *TRX*. Their stability under various treatments was evaluated using the Delta Ct method, as well as geNorm, NormFinder, BestKeeper, and RefFinder. The treatments encompassed different developmental stages, larval tissues, adult sexes, exposure to plant secondary substance stress, and insecticide applications. This research identifies suitable reference genes for functional gene studies under xenobiotic stress and establishes a foundation for exploring host adaptation and insecticide resistance mechanisms in *H. armigera*.

## 2. Materials and Methods

### 2.1. Insect Rearing

The cotton bollworm was reared under controlled conditions at a temperature of 27 ± 1 °C. The relative humidity was maintained at 50% during the larval stage and 70% during the adult stage, with a photoperiod of 14 h light and 10 h darkness. After eclosion, adults were transferred into mating cages. They were provided with fresh honey water daily and allowed to oviposit on gauze, which was replaced every 24 h. The gauze with eggs was then placed in rearing containers. Upon hatching, larvae were fed an artificial diet. The compositions of the artificial diet for larvae and the honey water for adults were based on the methods described by Liang [20].

The laboratory population of *H. armigera* was initiated from pupae (F0 generation) purchased from Keyun Biology (Baiyun Industry Co., Ltd., Jiyuan, China). Adults that emerged from these pupae were placed in mating cages to oviposit. Insects from the F1 generation were used as the source for experiments involving different developmental stages and tissues, while the sixth instar of the F2 generation was used for experiments involving stress induced by various plant secondary substances. The field population of *H. armigera* was collected as larvae (F0 generation) from a soybean field in the Shihezi area. The collected larvae were individually reared in tubes with the artificial diet. The third instar larvae of the F1 generation were used for experiments involving stress from different insecticide treatments.

### 2.2. Insect Treatments

For biotic factor treatments, 200 first-day eggs, 60 first-instar larvae, 30 s-instar larvae, 10 third-instar larvae, 8 fourth-instar larvae, 3 fifth-instar larvae, 3 sixth-instar larvae, 3 third-day pupae, 3 third-day female adults, 3 third-day male adults, and five tissues of 4 sixth-larvae (head, integument, midgut, hindgut, and fat body) were collected from the laboratory population for each replication. Each experimental condition had three biological replicates.

Tannic acid (98% purity, Yuanye, Shanghai, China), 2-tridecanone (98% purity, Macklin, Shanghai, China), and quercetin (97% purity, Macklin, Shanghai, China) were dissolved by 500 μL absolute ethanol, diluted with 500 μL distilled water, and then incorporated into the artificial diet at specific mass ratios, followed by thorough mixing. The final treatment concentrations were 0.25%, 1%, and 2% for 2-tridecanone; 0.05%, 0.1%, and 1% for tannic acid; and 0.1%, 0.5%, and 1% for quercetin. The blank control was treated with 1 mL of 50% ethanol. ZQ-8 (98% purity, a synthesized compound from the active substance of *Ferula samarcandica* (Apiales: Apiaceae) by the laboratory [21]) was dissolved in 100 μL dimethyl sulfoxide (DMSO) and then adjusted to 1 mL with 0.1% Triton X-100 solution. The solution was incorporated into the artificial diet at specific mass ratios, followed by thorough mixing. The final concentrations of ZQ-8 were 0.005%, 0.02%, and 0.05%. The blank control was treated with 1 mL of a solution (DMSO: 0.1% Triton X-100 = 1:9). Uniform sixth-instar larvae from the laboratory population were starved for 2 h and then fed the 1 g artificial diet supplemented with the respective plant secondary substances. Midgut tissues of three surviving larvae were collected as one sample on 4 h, 8 h, 20 h, and 28 h after the initiation of stress exposure, respectively, with three replicates per treatment.

Chlorantraniliprole (98% purity, Aladdin, Shanghai, China) and indoxacarb (98% purity, Yuanye, Shanghai, China) were dissolved in DMSO to prepare 100,000 mg/L stock solutions. These stock solutions were diluted serially with 0.1% Triton X-100 solution to five concentration gradients, which were 10,000 mg/L, 2000 mg/L, 400 mg/L, 80 mg/L, and 16 mg/L, respectively. Uniform third-instar larvae from the field population were treated onto the pronotum of the larva with 1 μL insecticide. After the agent had penetrated, larvae were fed common artificial diets. Each insecticide concentration was repeated three times. A 0.1% Triton X-100 solution was used as a blank control, and the mortality rate was required to be less than 20% after 72 h in the control. The toxicity equations and correlation coefficients (r) were calculated by SPSS 27.0 software using the Probit regression model after 48 h chlorantraniliprole and indoxacarb treatments. The equations were *y* = 0.504*x* − 1.119 (*r* = 0.975) for chlorantraniliprole and *y* = 0.744*x* − 1.709 (*r* = 0.993) for indoxacarb. Among the equations, the variables *x* and *y*, respectively, were base-10 logarithmic transformations of the concentration and probability transformations of the mortality rate. These stock solutions were diluted serially with 0.1% Triton X-100 solution to final concentrations of 15 mg/L and 40 mg/L, respectively, with sublethal concentrations LC_30_ (the concentration of insecticide when the probability is 0.3). The test insects and insecticide treatment methods were the same as above. Five surviving larvae were collected as one sample at 6 h, 15 h, 24 h, 48 h, and 72 h post-treatment, respectively, with three replicates per treatment.

### 2.3. RNA Extraction and cDNA Synthesis

All the samples were collected according to the above instructions and then ground in liquid nitrogen. Total RNA was extracted and treated with RNase-free DNase I using the MiniBEST Universal RNA Extraction Kit (Takara Biomedical Technology, Dalian, China) according to the manufacturer’s instructions. RNA concentration and purity were measured by the NanoDrop 2000c Spectrophotometers (Thermo Scientific, Waltham, MA, USA), and RNA integrity was estimated by 1.2% agarose gel electrophoresis. Subsequently, 500 ng total RNA was reverse-transcribed into first-strand cDNA (10 μL) using the EasyScript First-Strand cDNA Synthesis SuperMix (TRANGEN BIOTECH, Beijing, China) as per the provided protocol. The resulting cDNA samples were stored at −80 °C for later use.

### 2.4. Candidate Reference Genes Selection and Primer Design

Based on previously published literature on *H. armigera*, a total of eleven candidate reference genes were selected: *Actin*, *Tubulin*, *EF1-α*, *RPS3*, *RPS15*, *RPL27*, *RPL32*, *28S*, *GAPDH*, *SOD*, and *TRX*. The sequences of primers for these genes were also sourced from their respective references [18,19,22]. All primer sequences were synthesized by Sangon Biotechnology (Shanghai, China) (Table 1). Using female adult cDNA as a template, temperature gradient PCR was performed for all candidate reference gene primers following the instructions of the Premix ExTaq (Takara Biomedical Technology, Dalian, China). The annealing temperature range for each primer pair was assessed via 3% agarose gel electrophoresis, and the optimal annealing temperature for each primer set was selected.

### 2.5. Real-Time Quantitative PCR Assay

A 10-fold cDNA dilution series with three replicates per concentration was used to make standard curves for the estimation of amplification efficiency (E = (10^[−1/slope]^ − 1) × 100%) and the correlation coefficient (R^2^) [4]. qPCR was performed for all candidate reference genes using cDNA templates derived from the various treatments, following the manufacturer’s instructions for the PerfectStart Green qPCR SuperMix (TRANGEN BIOTECH, Beijing, China) on a LightCycle 96 Real-Time PCR System (Roche, Basel, Switzerland). The amplification conditions were as follows: 95 °C for 30 s, followed by 40 cycles of 95 °C for 10 s and 60 °C for 30 s. Melting curves were analyzed from 60 °C to 95 °C to confirm the primer specificity and lack of primer dimers. Each reaction was repeated three times.

### 2.6. Stability Analysis of Reference Genes

Algorithms GeNorm [23], Normfinder [24], BestKeeper [25], and comparative ΔCt methods [26] were used to evaluate the stability of these candidate reference genes. The raw Ct values were converted into relative quantities and imported into the GeNorm and Normfinder software. GeNorm calculates the expression stability measure (M) and analyzes the pairwise variation (V) for each candidate reference gene, then excludes the most unstable genes with the highest M-values, progressively. In addition, pairwise variation (V_n_/V_n+1_) determines the optimal number of reference genes for normalization, for which 0.15 was the recommended threshold. Normfinder calculates the expression stability value (SV) on the basis of intra- and inter-groups for each reference gene. The high expression stability of this gene is reflected by a low SV-value. BestKeeper uses the Ct data of all candidate reference genes to calculate the stability based on the standard deviation (SD), Pearson correlation coefficient (r), and coefficient of variation (CV). The most stable gene is the one with the lowest SD and CV values. The range of variation in SD should be below 1. The comparative ΔCT method, which compares the relative expression of pairwise genes within each sample, was used to select the optimal reference gene. RefFinder assigns weighted rankings to the candidate genes and combined the results from GeNorm, Normfinder, and BestKeeper [27,28]. NormFinder and GeNorm (https://seqyuan.shinyapps.io/seqyuan_prosper/, accessed on 17 November 2025), BestKeeper (https://www.gene-quantification.de/bestkeeper.html, accessed on 18 November 2025), and RefFinder (https://blooge.cn/RefFinder/, accessed on 18 November 2025).

### 2.7. Validation of Reference Genes

To evaluate the validity of the selected reference genes, *GADD45* expression levels were determined in different plant secondary substance and insecticide treatments. The transcript levels of *GADD45* were compared among the results when the best [NF (1)], the worst [NF (11)], and the optimal recommended combination of genes [NF (1-2)] and [NF (1-3)] were used as normalization [29]. The relative expression levels were calculated by the 2^−△△Ct^ method [30]. Statistical analyses were performed using IBM SPSS Statistics Version 26.0 (SPSS Inc., Chicago, IL, USA), and OriginPro Version 2021 (OriginLab, Northampton, MA, USA) was used for data visualization. The plant secondary substance treatments were analyzed by one-way ANOVA using Tukey HSD post hoc tests within different concentrations at the same treatment time, and two-way ANOVA to assess the main and interaction effects of two factors (treatment times and concentrations). The insecticide-treated experiments were analyzed by one-way ANOVA with Tukey HSD post hoc tests. Prior to all ANOVAs, assumptions of normality (Shapiro–Wilk test) and homogeneity of variances (Levene’s test) were verified. Statistical significance was defined as *p* < 0.05.

## 3. Results

### 3.1. Amplification Specificity and Efficiency of Candidate Reference Genes

The optimal annealing temperature for the primers of eleven candidate reference genes and the target gene *GADD45* was determined using temperature gradient PCR. An annealing temperature of 60 °C was used for *Actin*, *Tubulin*, *EF-1α*, *RPS3*, *RPS15*, *RPL27*, *RPL32*, *28S*, *SOD*, *TRX*, *GAPDH*, and *GADD45* during qPCR (Figure S1). The primer specificity of these twelve genes for qPCR was validated using a single sharp peak in the melting curve analysis and specific bands in the agarose gel electrophoresis analysis (Figure S2).

Amplification standard curves were plotted to determine the primer efficiencies for the twelve genes. The calculated efficiencies (E) were as follows: *Actin*, 97.09%; *Tubulin*, 99.96%; *EF-1α*, 91.99%; *RPS3*, 93.52%; *RPS15*, 90.48%; *RPL27*, 95.26%; *RPL32*, 92.86%; *28S*, 89.28%; *GAPDH*, 90.40%; *SOD*, 93.70%; *TRX*, 96.61%; and *GADD45*, 89.46%. The value of correlation coefficients (R^2^) for all genes was greater than 0.99. This indicates a strong linear correlation between the template cDNA concentration and the Ct value, demonstrating the accuracy and reliability of the standard curves (Table 1).

### 3.2. Expression Level Analysis of Candidate Reference Genes

**Figure 1:**
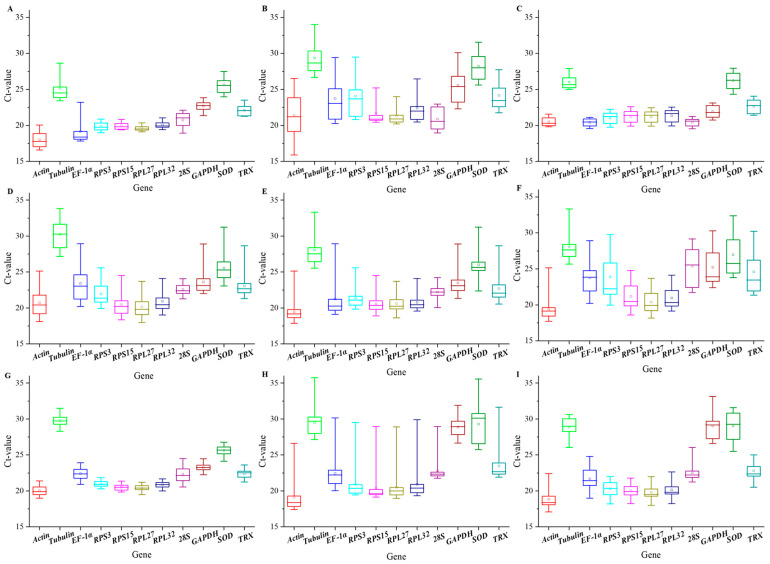
The Ct value distribution of eleven candidate reference genes across different experimental samples of *H. armigera*. (**A**) Development stages, (**B**) larval tissues of 6th instar, (**C**) adult sexes, (**D**) tannic-acid-treated 6th instar larvae, (**E**) quercetin-treated 6th instar larvae, (**F**) 2-tridecanone-treated 6th instar larvae, (**G**) ZQ-8-treated 6th instar larvae, (**H**) chlorantraniliprole-treated 3rd instar larvae, and (**I**) indoxacarb-treated 3rd instar larvae. The large outside box is determined from the 25th to 75th percentiles, and the line across the large box is the median. The small inside box represents the mean value. The solid line represents percentiles from 5th to 95th. Note: This figure only describes the expression levels of reference genes and does not perform statistical analysis on the Ct values.

The qPCR analysis revealed varying expression levels (indicated by Ct values) and stability across the eleven candidate reference genes under different experimental conditions. Except larval tissues, adult sexes, and tanni-acid-treated groups, the median Ct value of *Actin* was the least that means the highest expression level in all samples. The lowest expression was *Tubulin* in tissues, plant secondary substances, and insecticide-treated groups, whereas it was *SOD* in the stages and sexes groups. The greatest variation in expression was *EF-1α*, *Actin*, *SOD*, *EF-1α*, *EF-1α*, *RPS3*, *28S*, *RPL32*, and *GAPDH*, respectively, in stages, tissues, sexes, tannic acid, quercetin, 2-tridecanone, ZQ-8, chlorantraniliprole, and indoxacarb groups (Figure 1).

### 3.3. Stability of Candidate Reference Genes Under Biotic Conditions

#### 3.3.1. Developmental Stages

*Tubulin* was the least stable gene in the rankling of the four algorithms. The stability ranking of Normfinder was closely similar to that of the Delta Ct method, while *RPS3* was the most stable gene. *RPL27* was the most stable gene in the rankling of BestKeeper. The mean M value of *RPS15* and *RPL27* was the same as that determining the best genes in GeNorm. A comprehensive analysis using RefFinder generated the overall stability ranking as follows: *RPS3* > *RPL27* > *RPL32* > *RPS15* > *TRX* > *GAPDH* > *Actin* > *SOD* > *28S* > *EF-1α* > *Tubulin* (Table 2).

#### 3.3.2. Larval Tissues

The stability ranking recommended by Normfinder was closely similar to the results obtained from the Delta Ct method, which revealed *RPL32* to be the most stable gene. According to BestKeeper, only *RPL27* exhibited a standard deviation below 1 and a low coefficient of variation, suggesting it has stable expression. The most stable genes determined by GeNorm were *RPS15* and *RPL27*. A comprehensive ranking generated by RefFinder sorted the genes by stability as follows: *RPL32* > *RPL27* > *RPS15* > *TRX* > *GAPDH* > *SOD* > *Tubulin* > *28S* > *RPS3* > *EF-1α* > *Actin* (Table 2).

#### 3.3.3. Adult Sexes

The four computational programs, except BestKeeper, identified *Tubulin* as the least stable gene. *RPS3* and *RPL27* were the most stable genes in the stability ranking recommended by the Delta Ct method and GeNorm. *EF-1α* was the most stable gene from Normfinder. BestKeeper identified *EF-1α* and *28S* as the most stable genes. A comprehensive ranking generated by RefFinder sorted the genes by stability as follows: *RPS3* > *RPL27* > *EF-1α* > *Actin* > *RPS15* > *28S* > *RPL32* > *SOD* > *TRX* > *GAPDH* > *Tubulin* (Table 2).

### 3.4. Stability of Candidate Reference Genes Under Plant Secondary Substance Treatment

#### 3.4.1. Tannic Acid

The four computational programs identified *EF-1α* as the least stable gene. *GAPDH* was the most stable gene in the stability ranking recommended by the Delta Ct method and Normfinder. BestKeeper identified *28S* as the most stable gene, whereas those determined by GeNorm were *RPS15* and *RPL27*. According to RefFinder, the stability ranking of the reference genes from the most stable to the least stable across the tannic-acid-treated groups was: *GAPDH* > *RPL32* > *TRX* > *RPS15* > *RPL27* > *RPS3* > *28S* > *SOD* > *Actin* > *Tubulin* > *EF-1α* (Table 2).

#### 3.4.2. Quercetin

The Delta Ct method and Normfinder both determined *RPS3* as the most stable gene, while *28S* was the least stables gene. BestKeeper identified *28S* and *RPL32* to be the most stable genes. *RPL27* and *RPL32* were the best in the stability ranking of GeNorm. According to RefFinder, the stability ranking of the reference genes from the most stable to the least stable across the quercetin-treated groups was: *RPL32* > *RPS3* > *Actin* > *RPS15* > *RPL27* > *TRX* > *28S* > *GAPDH* > *SOD* > *Tubulin* > *EF-1α* (Table 2).

#### 3.4.3. 2-Tridecanone

*RPS15* and *28S*, respectively, were the most and least stable genes using the Delta Ct method, Normfinder, and GeNorm. BestKeeper determined *Actin* and *RPS3* as the most and least stable genes, respectively. According to RefFinder, the stability ranking of the reference genes from the most stable to the least stable across the 2-tridecanone-treated groups was: *RPS15* > *RPL32* > *RPL27* > *Actin* > *GAPDH* > *SOD* > *TRX* > *EF-1α* > *Tubulin* > *RPS3* > *28S* (Table 2).

#### 3.4.4. ZQ-8

All the four computational programs identified *28S* as the least stable gene. The stability rankings of the Delta Ct method and Normfinder were similar, for which *RPS3* was the most stable reference gene. The stability rankings of BestKeeper and GeNorm were similar, for which *RPL27* was the most stable reference gene. According to RefFinder, the stability ranking of the reference genes from the most stable to the least stable across the ZQ-8-treated groups was: *RPS3* > *RPL32* > *RPS15* > *RPL27* > *GAPDH* > *TRX* > *Actin* > *SOD* > *Tubulin* > *EF-1α* > *28S* (Table 2).

### 3.5. Stability of Candidate Reference Genes Under Insecticide Treatment

#### 3.5.1. Chlorantraniliprole

The least stable gene was *RPS3* in the rankings of the Delta Ct method, Normfinder, and GeNorm, while it was *SOD* in BestKeeper. The best reference gene was *TRX*, *28S*, *Tubulin*, and *RPL27*/*RPL32*, respectively, in the Delta Ct method, BestKeeper, Normfinder, and GeNorm. Using RefFinder for comprehensive analysis, the stability ranking of the candidate reference genes was: *RPL27* > *TRX* > *28S* > *RPL32* > *Tubulin* > *EF-1α* > *GAPDH* > *Actin* > *RPS15* > *SOD* > *RPS3* (Table 2).

#### 3.5.2. Indoxacarb

The same stability rankings were determined from the Delta Ct method, Normfinder, and GeNorm, for which the best was *RPL27* and the worst was *SOD*. The worst was *SOD* from BestKeeper, while the best was *28S*. Using RefFinder for comprehensive analysis, the stability ranking of the candidate reference genes was: *RPL27* > *RPL32* > *RPS15* > *TRX* > *28S* > *RPS3* > *Actin* > *EF-1α* > *Tubulin* > *GAPDH* > *SOD* (Table 2).

### 3.6. Combination of the Best Number of Reference Genes

**Figure 2:**
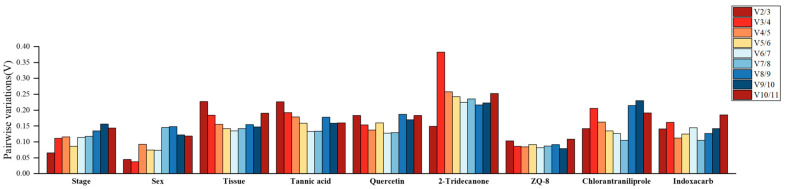
Pairwise variation (V) of candidate reference genes analyzed by GeNorm. Pairwise variation (V_n_/V_n+1_) was analyzed between the normalization factors (NF_n_ and NF_n+1_) by geNorm to determine the optimal number of reference genes. The V_n_/V_n+1_ values below 0.15 suggested that there was no need to introduce another gene.

This study failed to identify a candidate reference gene with constant expression in *H. armigera* under all the experimental conditions. So, the optimal number of reference genes was determined by the V_n/n+1_ value of GeNorm analysis for normalization (Figure 2). The V_2/3_ was below the proposed 0.15 cut-off value in stages, sexes, 2-tridecanone, ZQ-8, chlorantraniliprole, and indoxacarb groups, for which the two best selected reference genes were normalized in these groups. The values of V_5/6_, V_6/7_, and V_4/5_ were below 0.15 in tissues, tannic acid, and quercetin treatments, respectively, while it would be necessary to use three or more best reference genes for normalization.

### 3.7. Validation of Reference Gene Selection

**Figure 3:**
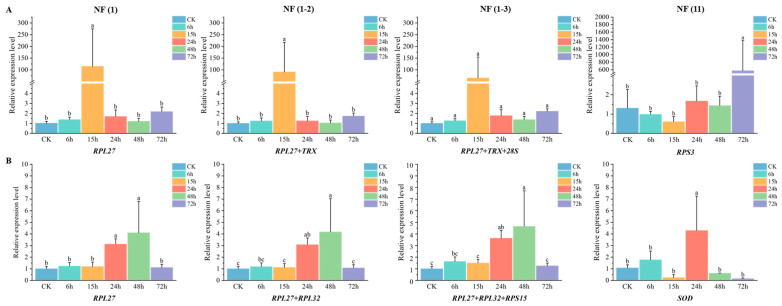
Effect of different reference genes to normalize the relative expression of the *GADD45* gene in (**A**) chlorantraniliprole-treated and (**B**) indoxacarb-treated 3rd instar larvae. NF (1), NF (1-2), NF (1-3), and NF (11) represent the most stable reference gene, the two most stable reference genes, the three most stable reference genes, and the least stable gene, respectively. Note: Different lowercase letters indicate significant differences (*p* < 0.05).

**Figure 4:**
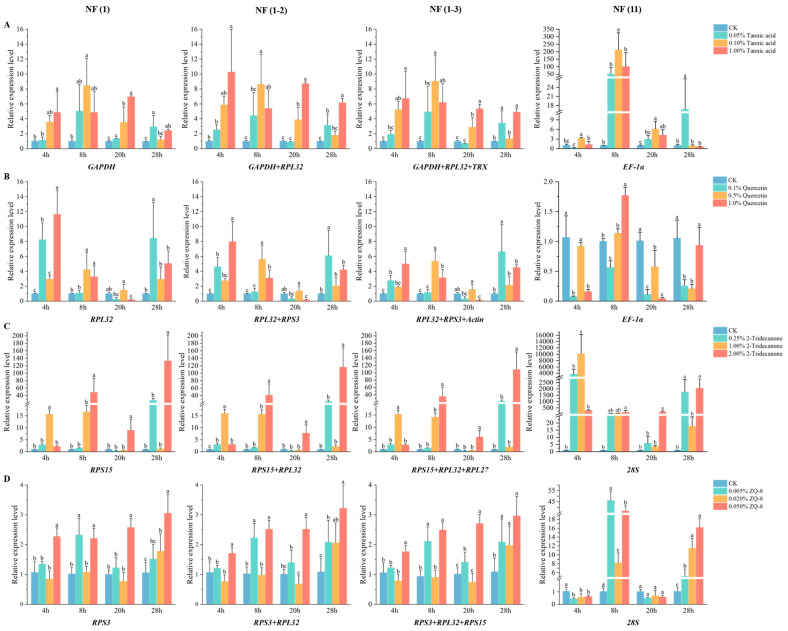
Effect of different reference genes to normalize the relative expression of the *GADD45* gene in different plant secondary substances exposed to 6th instar larvae. (**A**) Tannic acid, (**B**) quercetin, (**C**) 2-tridecanone, and (**D**) ZQ-8. NF (1), NF (1-2), NF (1-3), and NF (11) represent the most stable reference gene, the two most stable reference genes, the three most stable reference genes, and the least stable gene, respectively. Note: Different lowercase letters indicate significant differences (*p* < 0.05).

The expression level of the target gene *GADD45* was normalized under treatments involving various insecticides and plant secondary substances, using four distinct approaches. These included the most stable reference gene [NF (1)], the two most stable reference genes [NF (1-2)], the three most stable reference genes [NF (1-3)], and the least stable gene [NF (11)]. As illustrated in Figure 3 and Figure 4, the expression profile of *GADD45* remained consistent across all treatment groups when using [NF (1)], [NF (1-2)], or [NF (1-3)] as the reference. In contrast, the use of [NF (11)] as the reference yielded a completely different expression profile. Furthermore, the significance of [NF (1-3)] was distinct from [NF (1)] and [NF (1-2)] in chlorantraniliprole and ZQ-8 treatments. Based on the results of statistical analysis and the suggestion of MIQE 2.0 [4], the method employing the two most stable reference genes [NF (1-2)] was selected for calculating the relative expression of the target gene in this study.

### 3.8. Expression Profile of GADD45 Under Abiotic Conditions

Third-instar larvae were treated with sublethal doses of insecticides, and the relative expression level of the *GADD45* gene in the midgut was measured by qPCR using [NF (1-2)] as the reference. Compared with the control, *GADD45* expression was significantly upregulated 15 h after chlorantraniliprole treatment, as well as at 24 h and 48 h after indoxacarb treatment (Figure 3).

**Figure 5:**
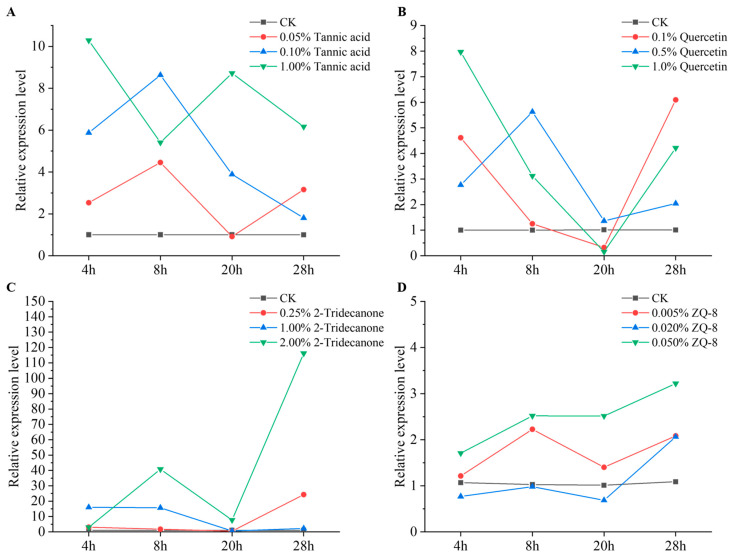
Expression profile of the *GADD45* gene under four different concentrations of plant secondary substances exposed to 6th instar larvae. (**A**) Tannic acid, (**B**) Quercetin, (**C**) 2-Tridecanone, (**D**) ZQ-8. The target gene was normalized using best combination of reference gene.

Sixth-instar larvae were exposed to varying concentrations of plant secondary metabolites, and the impact on *GADD45* gene expression was assessed using qPCR. Analysis of the results depicted in Figure 5A revealed a substantial influence of both tannic acid concentration and stress duration on *GADD45* expression (F_df3_ = 42.40, *p* < 0.0001 for concentration; F_df3_ = 3.34, *p* < 0.05 for time). The peak expression of *GADD45* occurred following a 4 h exposure to a high (1%) tannic acid concentration, registering a 10.24-fold increase compared to the control. Moreover, *GADD45* expression was significantly elevated after 8 h of treatment with moderate (0.1%) and low (0.05%) tannic acid concentrations. In Figure 5B, it was observed that the highest *GADD45* expression levels were recorded after a 4 h exposure to a high (1%) quercetin concentration, showing a 7.93-fold increase compared to the control. Similarly, a low (0.1%) quercetin concentration led to a significant rise in *GADD45* expression after 4 hr, while a moderate (0.5%) quercetin concentration exhibited a noticeable increase after 8 h. Both the quercetin concentration and stress duration had a significant impact on *GADD45* expression levels (F_df3_ = 18.96, *p* < 0.0001 for concentration; F_df3_ = 27.11, *p* < 0.0001 for time). The outcomes depicted in Figure 5C revealed that all three levels of 2-tridecanone triggered an upsurge in *GADD45* expression after 4 h, with the moderate concentration (1%) exhibiting the most pronounced effect. Notably, the elevated concentration (2%) led to the highest expression following 28 h of exposure to stress, peaking at 115.97 times that of the control. Both the concentration and duration of 2-tridecanone exposure exerted a significant impact on *GADD45* expression (F_df3_ = 48.15, *p* < 0.0001 for concentration; F_df3_ = 32.49, *p* < 0.0001 for time). Similarly, the data presented in Figure 5D demonstrated that both the concentration and duration of ZQ-8 treatment had a substantial influence on *GADD45* expression (F_df3_ = 45.19, *p* < 0.0001 for concentration; F_df3_ = 19.25, *p* < 0.0001 for time). Specifically, low (0.005%), moderate (0.02%), and high (0.05%) concentrations markedly induced *GADD45* overexpression after 8 h, 28 h, and 4 h of treatment, respectively. The most notable elevation in *GADD45* expression occurred after 28 h of treatment with the high concentration, reaching 2.96 times that of the control.

## 4. Discussion

Real-time quantitative PCR serves as a reliable method for analyzing gene expression. To mitigate expression variations among samples, it is essential to normalize the data using appropriate reference genes [4]. Numerous studies have examined the selection of reference genes in insects under various abiotic and biotic conditions. These investigations demonstrate that no single reference gene exhibits consistent expression levels across all experimental conditions within the same insect species, and homologous reference genes are not universally applicable among different insect species [31,32]. Therefore, it is crucial to identify suitable reference genes for quantitative studies tailored to specific conditions.

This study screened reference genes for the cotton bollworm under three biological factors: developmental stages, larval tissues, and adult sex (Table 2). The most suitable reference gene for developmental stages was *RPS3*, whereas *Tubulin* exhibited the least stability. *RPL27*, *RPL32*, and *RPS15* demonstrated relatively stable expression, in contrast to *EF1-α* and *28S*, which displayed unstable expression. These results diverge from those reported in three prior studies on reference genes across different developmental stages in cotton bollworm. Chandra et al. identified *Tubulin* as the most stable gene, and Shakeel et al. found *RPL28* and *RPS15* to be the two most stable reference genes, while the optimal combination of reference genes was *28S* and *RPS15* according to Zhang et al. in a separate study [17,18,19]. This discrepancy may arise from variations in population sources and specific instars of cotton bollworm, as the stability of reference genes can fluctuate based on genetic background and rearing history. In larval tissues, the study identified *RPL32*, *RPL27*, and *RPS15* as the most suitable reference genes. These findings are in close agreement with those of the top three genes, which were *RPS15*, *RPL13*, and *RPL32* according to Zhang [19]. The suitable reference genes were determined as *RPS3*, *RPL27*, and *EF1-α* for adult sex in our research. *EF1-α*, *RPL27*, and *RPS15* were reported as the top three genes for adult tissues in another paper [19]. Despite the difference in focus on sex versus tissues, both investigations utilized samples from adults without external interventions, leading to relatively consistent selections of reference genes.

This study was the first to explore the impact of various plant secondary substances on commonly used reference genes in the cotton bollworm, including tannic acid, quercetin, 2-tridecanone, and ZQ-8 (see Table 2). Tannic acid, a natural polyphenolic compound, is commonly present in plant vascular tissues [33]. Quercetin belongs to the flavonoid class and is distributed across a variety of vegetables, fruits, and spices [34]. 2-Tridecanone, a significant secondary substance in wild tomatoes, belongs to the group of volatile methyl ketones [35]. ZQ-8 is an artificial analog of Tschimganin 146 isolated from Ferula, classified as a terpenoid ester [21]. Despite their differences in type and source, all these substances share the common ability to hinder insect feeding, disrupt food digestion and utilization, and even induce insect poisoning or death [36,37,38,39]. Recent literature has also discussed the selection of internal references in insects for various host plants. In *Bemisia tabaci* (Hemiptera: Aleyrodidae), *HSP90*, *RPL29*, and *EF1-α* were identified as the most suitable reference genes across cabbage, tomato, and cucumber [5]. *RP49* and *RPL24* were found to be the most stable genes across both natural and artificial diets in *Maruca vitrata* (Lepidoptera: Crambidae) [40]. For *Spodoptera frugiperda* (Lepidoptera: Noctuidae), the optimal combination was found to be *18S*, *RPL10*, and *SOD* under different diets such as corn, rice, and wheat seedlings [41]. *RPS15* and *RPS26* were identified as the optimal reference genes in *Hyphantria cunea* (Lepidoptera: Erebidae) for various hosts including *Populus deltoides* (Salicales: Salicaceae), *Camptotheca acuminata* (Primulales: Nyssaceae), and *Cerasus serrulata* (Rosales: Rosaceae) [42]. The optimal combinations of reference genes varied after exposure to four plant secondary substances in our study. However, the expression levels of *RPL32* remained more consistent in the four groups, making it suitable for direct quantitative analysis.

Ribosomal proteins (RPs) are integral components of ribosomes, essential for protein biosynthesis in all cells, and are involved in DNA repair, cell differentiation, and regulating cell growth [43,44]. The *RPL27* was the most stable reference gene within two insecticide groups (see Table 2). In lepidopteran insects subjected to insecticide stress, several RP genes exhibit stable expression. For example, the combination of control genes recommended for Cry1Ac toxin was three genes: *EF1-α*, *RPS13*, and *RPL32* in *Plutella xylostella* (Lepidoptera: Plutellidae) [29]. *RPS15* and *RPL32* were sufficient to normalize gene expression in samples treated with *Bacillus thuringiensis* (Bt) (Bacillales: Bacillaceae) powder, beta-cypermethrin, and spinetoram of *H. armigera* [19]. Nevertheless, the stability of ribosomal protein genes is not universally applicable to all insects. In *Bombyx mori* (Lepidoptera: Bombycidae) exposed to fenvalerate and 2,2-dichlorovinyl dimethyl phosphate (DDVP), the *Actin*, *GAPDH*, and *Tubulin* were the most suitable reference genes [45]. It is noteworthy that while this study revealed RP genes as relatively stable in response to plant secondary substances and insecticides stressed, the recommendation of reference genes should be interpreted within each experimental condition. Cross-context comparisons could be influenced by variations in population or developmental stage.

This study failed to identify a candidate reference gene with constant expression in *H. armigera* under all the experimental conditions. Thus, the effect of using “wrong” reference genes for normalizing qPCR data must be determined. Growth arrest and DNA-damage-inducible gene 45 (*GADD45*), a pivotal gene involved in DNA damage repair, typically exhibits low expression levels under normal circumstances. However, exposure to external stressors can notably elevate its expression, serving as an early indicator of DNA damage [46]. The upregulated *GADD45* then activates the p38 and c-Jun N-terminal kinase (JNK) signaling pathways, leading to either cellular repair or apoptosis. This process is crucial for preserving genomic integrity, halting the cell cycle, and modulating apoptosis and immune reactions [47,48]. The study evaluated the expression patterns of the target gene *GADD45* in response to various plant secondary substances and insecticides, utilizing [NF (1)], [NF (1-2)], [NF (1-3)], and [NF (11)] as reference genes. The results indicated that [NF (1-2)] was the most suitable reference gene (see Figure 3 and Figure 4), supporting the consensus that employing multiple reference genes enhances the accuracy of qPCR. Notably, the relative expression level of *GADD45* in cotton bollworm increased following treatment with chlorantraniliprole, indoxacarb, 2-tridecanone, tannic acid, quercetin, and ZQ-8; however, the patterns of change for each treatment varied significantly.

## 5. Conclusions

The findings of this study highlight the critical need for selecting suitable reference genes to ensure accurate normalization in gene expression research. In particular, the optimal reference gene pairs were identified as follows: *GAPDH* + *RPL32*, *RPL32* + *RPS3*, *RPS15* + *RPL32*, *RPS3* + *RPL32*, *RPL27* + *TRX*, and *RPL27* + *RPL32*, in larvae of cotton bollworm exposed to tannic acid, quercetin, 2-tridecanone, ZQ-8, chlorantraniliprole, and indoxacarb. Additionally, larvae subjected to sublethal doses of plant secondary substances and insecticides demonstrated a significant upregulation of *GADD45* expression. This observation suggests that *GADD45* plays a role in the detoxification metabolic processes of cotton bollworm. This study establishes a reliable foundation for molecular research regarding the responses to plant secondary substances and insecticide stress, while also identifying potential novel target sites for managing insecticide resistance in *H. armigera*.

## Figures and Tables

**Table 1:** Candidate reference genes and target gene descriptions, primer sequences, and amplicon characteristics in this study.

Gene Symbol	Gene ID	Primer Sequence (5′–3′)	Size (bp)	PCR Efficiency (%)	Regression Coefficient (R^2^)
*Actin*	110378032	F: GACGGTCAGGTCATCACCATC	151	97.09	0.9951
		R: ACAGGTCCTTACGGATGTCA			
*Tubulin*	110379521	F: ATCAGAGAGGAATATCCC	151	99.96	0.9985
		R: CATTGTCGATACAGTAGG			
*EF-1α*	110372934	F: GACAAACGTACCATCGAGAAG	279	91.99	0.9972
		R: GATACCAGCCTCGAACTCAC			
*RPS3*	110373935	F: ACGGAGTTTTCAAGGCGGAA	208	93.52	0.9993
		R: GACTGCTCCGGGATGTTGAA			
*RPS15*	124636953	F: CCGAGATTGTTAAGACAC	152	90.48	0.9989
		R: GTATGTGACTGAGAACTC			
*RPL27*	110383711	F: ACAGGTATCCCCGCAAAGTGC	155	95.26	0.9988
		R: GTCCTTGGCGCTGAACTTCTC			
*RPL32*	126056134	F: CATCAATCGGATCGCTATG	152	92.86	0.9988
		R: CCATTGGGTAGCATGTGAC			
*28S*	135119273	F: CGATAGCGAACAAGTACCGT	100	89.28	0.9984
		R: TTCGAGTTTCGCAGGTTTAC			
*GAPDH*	110377691	F: CCAGAAGACAGTGGATGGAC	140	90.40	0.9997
		R: TACCAGTCAGCTTTCCGTTC			
*SOD*	110377870	F: CATGGATTCCATGTTCACGAG	132	93.70	0.9975
		R: GTTGCCGAGGTCTCCAACATG			
*TRX*	110382630	F: GTCGATCCACATCAAGGAC	140	96.61	0.9993
		R: CATTGGCCATCTCATCTAG			
*GADD45*	110381518	F: TCCAAGAACAGCAACCGA	167	89.46	0.9961
		R: CAGCAGCCGAGAAGTTTG			

**Table 2:** The stability ranking of candidate reference genes by Delta CT, BestKeeper, GeNorm, Normfinder, and RefFingder. SD [±Ct], standard deviation of the Ct; CV [%Ct], coefficient of variance expressed as a percentage of the Ct level.

Treatments	Rank	Delta CT		BestKeeper			GeNorm		Normfinder		RefFinder	
		Gene	Average of SD	Gene	SD [±Ct]	CV [%Ct]	Gene	Stability (*M*)	Gene	Stability	Gene	Stability
Development Stage	1	*RPS3*	0.82	*RPL27*	0.34	1.75	*RPS15*	0.155	*RPS3*	0.183	*RPS3*	2
	2	*RPL32*	0.86	*RPL32*	0.37	1.86	*RPL27*	0.155	*TRX*	0.186	*RPL27*	2.34
	3	*TRX*	0.86	*RPS15*	0.4	2.02	*RPL32*	0.184	*GAPDH*	0.338	*RPL32*	2.91
	4	*GAPDH*	0.91	*RPS3*	0.6	3.03	*RPS3*	0.319	*RPL32*	0.455	*RPS15*	3.08
	5	*RPS15*	0.92	*GAPDH*	0.69	3.06	*TRX*	0.434	*RPS15*	0.553	*TRX*	3.16
	6	*RPL27*	0.92	*TRX*	0.69	3.14	*GAPDH*	0.484	*RPL27*	0.568	*GAPDH*	4.56
	7	*Actin*	1.24	*SOD*	0.86	3.37	*Actin*	0.596	*Actin*	1.011	*Actin*	7.45
	8	*SOD*	1.28	*28S*	1	4.8	*SOD*	0.706	*SOD*	1.014	*SOD*	7.74
	9	*28S*	1.42	*Actin*	1.04	5.77	*28S*	0.842	*28S*	1.164	*28S*	8.74
	10	*EF-1α*	1.58	*EF-1α*	1.31	6.84	*EF-1α*	1.01	*EF-1α*	1.4	*EF-1α*	10
	11	*Tubulin*	1.73	*Tubulin*	1.37	5.46	*Tubulin*	1.14	*Tubulin*	1.591	*Tubulin*	11
Larval Tissue	1	*RPL32*	1.05	*RPL27*	0.98	4.57	*RPS15*	0.479	*RPL32*	0.228	*RPL32*	1.86
	2	*TRX*	1.09	*RPS15*	1.31	6.04	*RPL27*	0.479	*TRX*	0.252	*RPL27*	2.83
	3	*GAPDH*	1.13	*28S*	1.44	6.89	*RPL32*	0.643	*GAPDH*	0.446	*RPS15*	3.03
	4	*Tubulin*	1.18	*RPL32*	1.56	6.96	*TRX*	0.732	*Tubulin*	0.561	*TRX*	3.13
	5	*SOD*	1.22	*SOD*	1.63	5.76	*SOD*	0.798	*SOD*	0.693	*GAPDH*	4.74
	6	*RPS15*	1.32	*TRX*	1.65	6.84	*Tubulin*	0.859	*RPS3*	0.939	*SOD*	5
	7	*RPS3*	1.34	*Tubulin*	1.86	6.32	*GAPDH*	0.916	*RPS15*	0.945	*Tubulin*	5.09
	8	*RPL27*	1.4	*GAPDH*	2.13	8.33	*RPS3*	1.002	*RPL27*	1.106	*28S*	7.4
	9	*EF-1α*	1.56	*RPS3*	2.37	9.86	*EF-1α*	1.09	*EF-1α*	1.291	*RPS3*	7.42
	10	*28S*	1.79	*EF-1α*	2.75	11.59	*28S*	1.199	*28S*	1.589	*EF-1α*	9.24
	11	*Actin*	2.28	*Actin*	2.83	13.25	*Actin*	1.396	*Actin*	2.133	*Actin*	11
Adult Sex	1	*RPS3*	0.74	*EF-1α*	0.45	2.22	*RPS3*	0.045	*EF-1α*	0.121	*RPS3*	1.86
	2	*RPL27*	0.74	*28S*	0.45	2.18	*RPL27*	0.045	*Actin*	0.263	*RPL27*	2.51
	3	*RPS15*	0.76	*Actin*	0.54	2.62	*RPS15*	0.078	*RPS3*	0.439	*EF-1α*	2.78
	4	*EF-1α*	0.79	*RPS3*	0.68	3.25	*RPL32*	0.107	*RPL27*	0.459	*Actin*	3.66
	5	*RPL32*	0.79	*RPL27*	0.7	3.32	*Actin*	0.256	*RPS15*	0.496	*RPS15*	4.05
	6	*Actin*	0.8	*RPS15*	0.73	3.43	*EF-1α*	0.336	*RPL32*	0.55	*28S*	4.76
	7	*SOD*	1.01	*RPL32*	0.78	3.64	*SOD*	0.402	*SOD*	0.83	*RPL32*	5.09
	8	*28S*	1.2	*Tubulin*	0.79	3.05	*28S*	0.605	*28S*	0.912	*SOD*	7.84
	9	*GAPDH*	1.37	*GAPDH*	0.83	3.8	*TRX*	0.792	*TRX*	1.209	*TRX*	9.24
	10	*TRX*	1.37	*TRX*	0.91	4.01	*GAPDH*	0.902	*GAPDH*	1.211	*GAPDH*	9.74
	11	*Tubulin*	1.47	*SOD*	1.07	4.09	*Tubulin*	1.006	*Tubulin*	1.297	*Tubulin*	10.16
Tannic Acid	1	*GAPDH*	1.11	*28S*	0.68	3.03	*RPS15*	0.336	*GAPDH*	0.38	*GAPDH*	2.11
	2	*RPL32*	1.14	*TRX*	1.16	5.02	*RPL27*	0.336	*RPS3*	0.491	*RPL32*	2.91
	3	*RPS3*	1.15	*RPL32*	1.17	5.6	*RPL32*	0.504	*TRX*	0.572	*TRX*	3.46
	4	*TRX*	1.19	*GAPDH*	1.22	5.16	*SOD*	0.642	*RPL32*	0.595	*RPS15*	3.5
	5	*RPS15*	1.24	*RPS15*	1.27	6.19	*GAPDH*	0.777	*Actin*	0.838	*RPL27*	3.98
	6	*RPL27*	1.29	*RPL27*	1.33	6.59	*TRX*	0.864	*RPS15*	0.873	*RPS3*	4.14
	7	*Actin*	1.32	*RPS3*	1.36	6.21	*RPS3*	0.923	*RPL27*	0.972	*28S*	5.62
	8	*SOD*	1.42	*SOD*	1.43	5.58	*Actin*	0.991	*SOD*	1.121	*SOD*	6.73
	9	*Tubulin*	1.68	*Actin*	1.55	7.47	*Tubulin*	1.147	*Tubulin*	1.375	*Actin*	7.09
	10	*28S*	1.86	*Tubulin*	1.55	5.14	*28S*	1.274	*28S*	1.6	*Tubulin*	9.24
	11	*EF-1α*	1.94	*EF-1α*	1.98	8.44	*EF-1α*	1.395	*EF-1α*	1.747	*EF-1α*	11
Quercetin	1	*RPS3*	1.07	*RPL32*	0.83	3.99	*RPL27*	0.505	*RPS3*	0.186	*RPL32*	2.06
	2	*Actin*	1.16	*28S*	0.83	3.72	*RPL32*	0.505	*Actin*	0.477	*RPS3*	2.11
	3	*RPL32*	1.18	*RPS15*	0.86	4.16	*RPS15*	0.571	*RPL32*	0.615	*Actin*	3.31
	4	*RPS15*	1.21	*RPL27*	1	4.84	*RPS3*	0.633	*TRX*	0.624	*RPS15*	3.66
	5	*TRX*	1.25	*RPS3*	1.02	4.75	*Actin*	0.689	*RPS15*	0.635	*RPL27*	3.74
	6	*GAPDH*	1.28	*Actin*	1.03	5.3	*GAPDH*	0.809	*GAPDH*	0.699	*TRX*	5.79
	7	*RPL27*	1.29	*GAPDH*	1.22	5.18	*TRX*	0.871	*RPL27*	0.854	*28S*	6.04
	8	*SOD*	1.51	*TRX*	1.36	6	*SOD*	0.949	*SOD*	1.151	*GAPDH*	6.24
	9	*Tubulin*	1.82	*SOD*	1.39	5.35	*Tubulin*	1.139	*Tubulin*	1.544	*SOD*	8.24
	10	*EF-1α*	1.98	*Tubulin*	1.69	6.03	*EF-1α*	1.282	*EF-1α*	1.788	*Tubulin*	9.24
	11	*28S*	2.19	*EF-1α*	1.89	8.88	*28S*	1.448	*28S*	2.004	*EF-1α*	10.24
2-Tridecanone	1	*RPS15*	1.57	*Actin*	0.96	4.94	*RPS15*	0.317	*RPS15*	0.396	*RPS15*	1.5
	2	*RPL32*	1.59	*RPL32*	1.25	5.97	*RPL27*	0.317	*RPL32*	0.527	*RPL32*	2.21
	3	*RPL27*	1.6	*RPL27*	1.38	6.78	*RPL32*	0.418	*RPL27*	0.598	*RPL27*	2.28
	4	*GAPDH*	1.87	*Tubulin*	1.39	4.94	*SOD*	0.982	*GAPDH*	1.112	*Actin*	4.12
	5	*SOD*	1.91	*EF-1α*	1.56	6.57	*GAPDH*	1.174	*SOD*	1.284	*GAPDH*	5.03
	6	*Actin*	2.03	*RPS15*	1.56	7.34	*TRX*	1.306	*Actin*	1.474	*SOD*	5.48
	7	*TRX*	2.12	*28S*	2.32	9.16	*RPS3*	1.439	*TRX*	1.653	*TRX*	7.36
	8	*EF-1α*	2.24	*GAPDH*	2.33	9.23	*Actin*	1.581	*EF-1α*	1.684	*EF-1α*	7.67
	9	*RPS3*	2.34	*SOD*	2.47	9.17	*EF-1α*	1.719	*RPS3*	1.941	*Tubulin*	7.95
	10	*Tubulin*	2.59	*TRX*	2.7	10.96	*Tubulin*	1.864	*Tubulin*	2.265	*RPS3*	8.89
	11	*28S*	3.08	*RPS3*	2.75	11.5	*28S*	2.086	*28S*	2.757	*28S*	9.82
ZQ-8	1	*RPS3*	0.61	*RPS15*	0.36	1.74	*RPL27*	0.239	*RPS3*	0.192	*RPS3*	2
	2	*RPL32*	0.62	*RPL27*	0.36	1.77	*RPL32*	0.239	*GAPDH*	0.243	*RPL32*	2.21
	3	*RPS15*	0.63	*RPS3*	0.38	1.82	*RPS15*	0.287	*RPS15*	0.256	*RPS15*	2.28
	4	*GAPDH*	0.64	*RPL32*	0.38	1.82	*RPS3*	0.333	*RPL32*	0.265	*RPL27*	2.66
	5	*RPL27*	0.65	*GAPDH*	0.46	1.96	*GAPDH*	0.386	*RPL27*	0.357	*GAPDH*	3.76
	6	*Actin*	0.77	*TRX*	0.55	2.44	*TRX*	0.458	*Actin*	0.493	*TRX*	6.48
	7	*TRX*	0.83	*Actin*	0.57	2.84	*SOD*	0.509	*TRX*	0.675	*Actin*	6.7
	8	*SOD*	0.88	*SOD*	0.62	2.42	*Actin*	0.57	*Tubulin*	0.726	*SOD*	7.97
	9	*EF-1α*	0.91	*Tubulin*	0.71	2.39	*Tubulin*	0.641	*SOD*	0.728	*Tubulin*	8.97
	10	*Tubulin*	0.92	*EF-1α*	0.73	3.25	*EF-1α*	0.687	*EF-1α*	0.73	*EF-1α*	9.74
	11	*28S*	1.28	*28S*	0.9	4.05	*28S*	0.795	*28S*	1.187	*28S*	11
Chlorantraniliprole	1	*TRX*	1.53	*28S*	0.96	4.21	*RPL27*	0.161	*Tubulin*	0.721	*RPL27*	2.21
	2	*RPL27*	1.54	*GAPDH*	1.09	3.76	*RPL32*	0.161	*28S*	0.841	*TRX*	2.71
	3	*RPL32*	1.6	*RPL27*	1.19	5.81	*TRX*	0.302	*TRX*	0.841	*28S*	3.03
	4	*Tubulin*	1.64	*RPS15*	1.22	5.97	*EF-1α*	0.568	*RPL27*	0.924	*RPL32*	3.08
	5	*EF-1α*	1.64	*RPL32*	1.25	5.97	*Tubulin*	0.737	*EF-1α*	0.961	*Tubulin*	3.56
	6	*28S*	1.69	*TRX*	1.29	5.51	*Actin*	0.842	*RPL32*	1.051	*EF-1α*	5.48
	7	*Actin*	1.75	*RPS3*	1.32	6.29	*28S*	0.916	*Actin*	1.073	*GAPDH*	5.66
	8	*GAPDH*	2.09	*Tubulin*	1.39	4.7	*GAPDH*	1.14	*GAPDH*	1.36	*Actin*	7.36
	9	*SOD*	2.51	*EF-1α*	1.41	6.29	*SOD*	1.369	*SOD*	2.08	*RPS15*	7.95
	10	*RPS3*	3.12	*Actin*	1.46	7.64	*RPS15*	1.777	*RPS15*	2.912	*SOD*	9.46
	11	*RPS15*	3.12	*SOD*	2.57	8.76	*RPS3*	2.021	*RPS3*	2.915	*RPS3*	9.82
Indoxacarb	1	*RPL27*	0.88	*28S*	0.73	3.23	*RPL27*	0.13	*RPL27*	0.065	*RPL27*	1.32
	2	*RPL32*	0.9	*RPS15*	0.74	3.72	*RPL32*	0.13	*RPL32*	0.138	*RPL32*	2
	3	*TRX*	0.95	*RPL27*	0.74	3.76	*TRX*	0.276	*TRX*	0.252	*RPS15*	3.36
	4	*RPS15*	1.05	*RPL32*	0.81	4.02	*RPS15*	0.469	*RPS15*	0.569	*TRX*	3.57
	5	*RPS3*	1.08	*RPS3*	0.87	4.28	*RPS3*	0.538	*RPS3*	0.606	*28S*	3.83
	6	*28S*	1.2	*TRX*	0.92	4.05	*28S*	0.637	*28S*	0.839	*RPS3*	5
	7	*Actin*	1.29	*Actin*	1.06	5.63	*Actin*	0.744	*Actin*	0.943	*Actin*	7
	8	*EF-1α*	1.31	*Tubulin*	1.12	3.87	*EF-1α*	0.843	*EF-1α*	0.961	*EF-1α*	8.24
	9	*Tubulin*	1.41	*EF-1α*	1.2	5.55	*Tubulin*	0.938	*Tubulin*	1.078	*Tubulin*	8.74
	10	*GAPDH*	1.6	*GAPDH*	1.38	4.76	*GAPDH*	1.051	*GAPDH*	1.343	*GAPDH*	10
	11	*SOD*	2.21	*SOD*	1.79	6.16	*SOD*	1.262	*SOD*	2.076	*SOD*	11

Note: The Rank from 1 to 11 indicates the stability of the reference gene from most to least stable.

## Data Availability

The original data are included in this article; further inquiries can be directed to the corresponding author.

## Supplementary Materials

The following supporting information can be downloaded at: https://www.mdpi.com/article/10.3390/biology15020175/s1. Figure S1: The annealing temperature analysis of qPCR amplification of eleven candidate genes and one target gene in *H. armigera*. Line 1–8 represent 51.6, 53.1, 54.9, 56.6, 58.2, 60.3, 62.0, and 63.8 °C of *Actin*, *Tubulin*, *EF-1α*, *RPS3*, *RPS15*, *RPL27*, *RPL32*, *28S*, *TRX*, and *GADD45* genes. Line 1–11 represent 51.2, 52.8, 54.2, 55.5, 56.9, 58.0, 59.2, 60.3, 61.8, 63.0, and 63.8 °C of *GAPDH* and *SOD* genes. Line n represents the negative control. Line M represents DL500 DNA Marker (Takara). Figure S2: The melt curve analysis of qPCR amplification of eleven candidate genes and one target gene in *H. armigera*, including *Actin*, *Tubulin*, *EF-1α*, *RPS3*, *RPS15*, *RPL27*, *RPL32*, *28S*, *GAPDH*, *SOD*, *TRX*, and *GADD45*.

## Abbreviations

The following abbreviations are used in this manuscript:

qPCR	Real-time quantitative polymerase chain reaction
LC30	Lethal Concentration for 30% of the individuals
DMSO	Dimethyl sulfoxide
Ct	Cycle threshold
R^2^	Correlation coefficients
E	Efficiencies
Tubulin	Beta-tubulin 1 chain
GAPDH	Glyceraldehyde-3-phosphate dehydrogenase
28S	28S ribosomal RNA
RPL32	Ribosomal protein L gene 32
RPL27	Ribosomal protein L gene 27
RPS15	Ribosomal protein S gene 15
RPS3	Ribosomal protein S gene 3
SOD	Cu/Zn superoxide dismutase
TRX	Thioredoxin
EF1-α	Elongation factor 1 alpha
GADD45	Growth arrest and DNA-damage-inducible gene 45

## References

[1] Nonis A., De Nardi B., Nonis A. (2014). Choosing between RT-qPCR and RNA-seq: A back-of-the-envelope estimate towards the definition of the break-even-point. Anal. Bioanal. Chem..

[2] Bustin S.A., Beaulieu J.F., Huggett J., Jaggi R., Kibenge F.S.B., Olsvik P.A., Penning L.C., Toegel S. (2010). MIQE précis: Practical implementation of minimum standard guidelines for fluorescence-based quantitative real-time PCR experiments. BMC Mol. Biol..

[3] Praité A., Lambert J.M., Delpy L., Al Hayek S. (2025). Guidelines for RNA analysis by reverse transcription quantitative polymerase chain reaction. Methods Mol. Biol..

[4] Bustin S.A., Ruijter J.M., van den Hoff M.J.B., Kubista M., Pfaffl M.W., Shipley G.L., Tran N., Rödiger S., Untergasser A., Mueller R. (2025). MIQE 2.0: Revision of the minimum information for publication of quantitative real-time PCR experiments guidelines. Clini. Chem..

[5] Li R.M., Xie W., Wang S.L., Wu Q.J., Yang N., Yang X., Pan H.P., Zhou X.M., Bai L.Y., Xu B.Y. (2013). Reference gene selection for qRT-PCR analysis in the sweetpotato whitefly, *Bemisia tabaci* (Hemiptera: Aleyrodidae). PLoS ONE.

[6] Jeon J.H., Moon K.H., Kim Y.H., Kim Y.H. (2020). Reference gene selection for qRT-PCR analysis of season- and tissue-specific gene expression profiles in the honey bee *Apis mellifera*. Sci. Rep..

[7] Zhou T., Feng H.H., Zhang J., Tang Y.L., Dong X.L., Kang K. (2025). Selection of *Sclerodermus pupariae* reference genes for quantitative real-time PCR. Insects.

[8] Zhang H.Z., Bleiker K.P., Keeling C.I., Liu Y.Z., Wei H.L., Zhang B., Han C.K., Erbilgin N. (2025). Reference genes selection for qRT-PCR analysis in *Dendroctonus rufipennis*. Gene.

[9] Shakeel M., Rodriguez A., Tahir U.B., Jin F.L. (2018). Gene expression studies of reference genes for quantitative real-time PCR: An overview in insects. Biotechnol. Lett..

[10] Lü J., Yang C.X., Zhang Y.J., Pan H.P. (2018). Selection of reference genes for the normalization of RT-qPCR data in gene expression studies in insects: A systematic review. Front. Physiol..

[11] Cunningham J.P., Zalucki M.P. (2014). Understanding heliothine (Lepidoptera: Heliothinae) pests: What is a host plant?. J. Econ. Entomol..

[12] Wang Z.G., Jiang S.S., Mota-Sanchez D., Wang W., Li X.R., Gao Y.L., Lu X.P., Yang X.Q. (2019). Cytochrome P450-mediated λ-cyhalothrinresistance in a field strain of *Helicoverpa armigera* from northeast China. J. Agric. Food Chem..

[13] Wu K.M., Guo Y.Y. (2005). The evolution of cotton pest management practices in China. Annu. Rev. Entomol..

[14] Wu C.F., Ding C.H., Chen S., Wu X.Y., Zhang L.Q., Song Y.Y., Li W., Zeng R.S. (2021). Exposure of *Helicoverpa armigera* larvae to plant volatile organic compounds induces cytochrome P450 monooxygenases and enhances larval tolerance to the insecticide methomyl. Insects.

[15] Lu Y.H. (2021). Ever-evolving advances in the researches of cotton insect pest management in China. J. Plant Prot..

[16] Huang Y., Wu P.Z., Zheng J.Y., Zhang Y., Qiu L.H. (2022). Status of resistance to chemical insecticides in cotton bollworm *Helicoverpa armigera* and research progresses on the molecular mechanisms. J. Plant Prot..

[17] Chandra G.S., Asokan R., Manamohan M., Krishna Kumar N.K., Sita T. (2014). Evaluation of reference genes for quantitative real-time PCR normalization in cotton bollworm, *Helicoverna armigera*. Mol. Biol..

[18] Shakeel M., Zhu X., Kang T.H., Wan H., Li J.H. (2015). Selection and evaluation of reference genes for quantitative gene expression studies in cotton bollworm, *Helicoverpa armigera* (Lepidoptera: Noctuidae). J. Asia-Pac. Entomol..

[19] Zhang S.D., An S.H., Li Z., Wu F.M., Yang Q.P., Liu Y.C., Cao J.J., Zhang H.J., Zhang Q.W., Liu X.X. (2015). Identification and validation of reference genes for normalization of gene expression analysis using qRT-PCR in *Helicoverpa armigera* (Lepidoptera: Noctuidae). Gene.

[20] Liang G.M., Tan W.J., Guo Y.Y. (1999). Improvement of the artificial rearing technology of cotton bollworms. J. Plant Prot..

[21] Zhou Y.T., Wang C.J., Xin F., Han X.Q., Zhang J., Sun K. (2018). Synthesis, insecticidal, fungicidal activities and structure-activity relationships of Tschimganin analogs. Molecules.

[22] Xing L.S., Yuan C.F., Wang M.L., Lin Z., Shen B.C., Hu Z.H., Zou Z. (2017). Dynamics of the interaction between cotton bollworm *Helicoverpa armigera* and nucleopolyhedrovirus as revealed by integrated transcriptomic and proteomic analyses. Mol. Cell. Proteom..

[23] Vandesompele J., De Preter K., Pattyn F., Poppe B., Van Roy N., De Paepe A., Speleman F. (2002). Accurate normalization of real-time quantitative RT-PCR data by geometric averaging of multiple internal control genes. Genome Biol..

[24] Andersen C.L., Jensen J.L., Ørntoft T.F. (2004). Normalization of real-time quantitative reverse transcription-PCR data: A model-based variance estimation approach to identify genes suited for normalization, applied to bladder and colon cancer data sets. Cancer Res..

[25] Pfaffl M.W., Tichopad A., Prgomet C., Neuvians T.P. (2004). Determination of stable housekeeping genes, differentially regulated target genes and sample integrity: BestKeeper-Excel-based tool using pair-wise correlations. Biotechnol. Lett..

[26] Silver N., Best S., Jiang J., Thein S.L. (2006). Selection of housekeeping genes for gene expression studies in human reticulocytes using real-time PCR. BMC Mol. Biol..

[27] Xie F.L., Xiao P., Chen D.L., Xu L., Zhang B.H. (2012). miRDeepFinder: A miRNA analysis tool for deep sequencing of plant small RNAs. Plant Mol. Biol..

[28] Xie F.L., Wang J.Y., Zhang B.H. (2023). RefFinder: A web-based tool for comprehensively analyzing and identifying reference genes. Funct. Integr. Genom..

[29] Fu W., Xie W., Zhang Z., Wang S.L., Wu Q.J., Liu Y., Zhou X.M., Zhou X.G., Zhang Y.J. (2013). Exploring valid reference genes for quantitative real-time PCR analysis in *Plutella xylostella* (Lepidoptera: Plutellidae). Int. J. Biol. Sci..

[30] Livak K.J., Schmittgen T.D. (2001). Analysis of relative gene expression data using realtime quantitative PCR and the 2^−△△CT^ method. Methods.

[31] Teng X.L., Zhang Z., He G.L., Yang L.W., Li F. (2012). Validation of reference genes for quantitative expression analysis by real-time RT-PCR in four Lepidopteran insects. J. Insect. Sci..

[32] Yang Y.H., Li Z., Cao J.J., Li Y.R., Li H., Yang Q.P., Zhang Q.W., Liu X.X. (2017). Identification and evaluation of suitable reference genes for normalization of MicroRNA expression in *Helicoverpa armigera* (lepidoptera: Noctuidae) using quantitative real-time PCR. J. Insect. Sci..

[33] Tang F., Tu H.Z., Shang Q.L., Gao X.W., Liang P. (2020). Molecular cloning and characterization of five glutathione S-transferase genes and promoters from *Micromelalopha troglodyta* (Graeser) (Lepidoptera: Notodontidae) and their response to tannic acid stress. Insects.

[34] Gao X.W., Dong X.L., Zhao Y., Zhen B.Z. (1999). Induction of carboxylesterase, glutathione S-transferase and acetylcholinesterase by quercetin in *Helicoverpa armigera*. Chin. J. Pest. Sci..

[35] Antonious G.F. (2001). Production and quantification of methyl ketones in wild tomato accessions. J. Environ. Sci. Health Part B.

[36] Zhang L., Lu Y., Xiang M., Shang Q.L., Gao X.W. (2016). The retardant effect of 2-Tridecanone, mediated by Cytochrome P450, on the development of cotton bollworm, *Helicoverpa armigera*. BMC Genom..

[37] Ma K.S., Tang Q.L., Liang P.Z., Xia J., Zhang B.Z., Gao X.W. (2019). Toxicity and sublethal effects of two plant allelochemicals on the demographical traits of cotton aphid, *Aphis gossypii* Glover (Hemiptera: Aphididae). PLoS ONE.

[38] Riddick E.W. (2021). Potential of quercetin to reduce herbivory without disrupting natural enemies and pollinators. Agriculture.

[39] Yang L., Chen M.H., Han X.Q., Liu C.Y., Wang C.J., Zhang G.Q., Yang D.S., Zhao S.F. (2022). Discovery of ZQ-8, a novel starting point to develop inhibitors against the potent molecular target Chitinase. J. Agric. Food Chem..

[40] Choudhury A., Verma S., Muthamilarasan M., Rajam M.V. (2021). Identification of suitable reference genes for expression profiling studies using qRT-PCR in an important insect pest, *Maruca vitrata*. Mol. Biol. Rep..

[41] Han S.P., Qin Q.J., Wang D., Zhou Y.Y., He Y.Z. (2021). Selection and evaluation of reference genes for qRT-PCR in *Spodoptera frugiperda* (Lepidoptera: Noctuidae). Insects.

[42] Zhao X.D., Geng Y.S., Hu T.Y., Zhao Y.G., Yang S.L., Hao D.J. (2022). Evaluation of optimal reference genes for qRT-PCR analysis in *Hyphantria cunea* (Drury). Insects.

[43] Zhou X., Liao W.J., Liao J.M., Liao P., Hua L. (2015). Ribosomal proteins: Functions beyond the ribosome. J. Mol. Cell Biol..

[44] Rambaldelli G., Bacci L., Pollutri D., Filipek K., Penzo M. (2025). Master of disguise: Ribosomal protein L5 beyond translation. Biochimie.

[45] Zhao Z.Q., Zheng K.Y., Ou Q., Xu P.Z., Qin S., Sun X., Li M.W., Wu Y.C., Wang X.Y. (2022). Identification of optimal reference genes in *Bombyx mori* (Lepidoptera) for normalization of stress-responsive genes after challenge with pesticides. Arch. Insect Biochem. Physiol..

[46] Tran H., Brunet A., Grenier J.M., Datta S.R., Fornace A.J., DiStefano P.S., Chiang L.W., Greenberg M.E. (2002). DNA repair pathway stimulated by the forkhead transcription factor FOXO3a through the Gadd45 protein. Science.

[47] Barreto G., Schäfer A., Marhold J., Stach D., Swaminathan S.K., Handa V., Döderlein G., Maltry N., Wu W., Lyko F. (2007). Gadd45α promotes epigenetic gene activation by repair-mediated DNA demethylation. Nature.

[48] Schäfer A. (2013). Gadd45 proteins: Key players of repair-mediated DNA demethylation. Adv. Exp. Med. Biol..

