# Supplementary material for: Identification and Stability Assessment of Reference Genes in Helicoverpa armigera Under Plant Secondary Substance and Insecticide Stresses
Source: Biology (Basel). 2026 Jan 17;15(2):175. doi: 10.3390/biology15020175 (PMC12837964; doi:10.3390/biology15020175)
Supplement: Supplementary file 1 [file biology-15-00175-s001.zip › biology-4088498-supplementary.pdf]

Supplementary materials

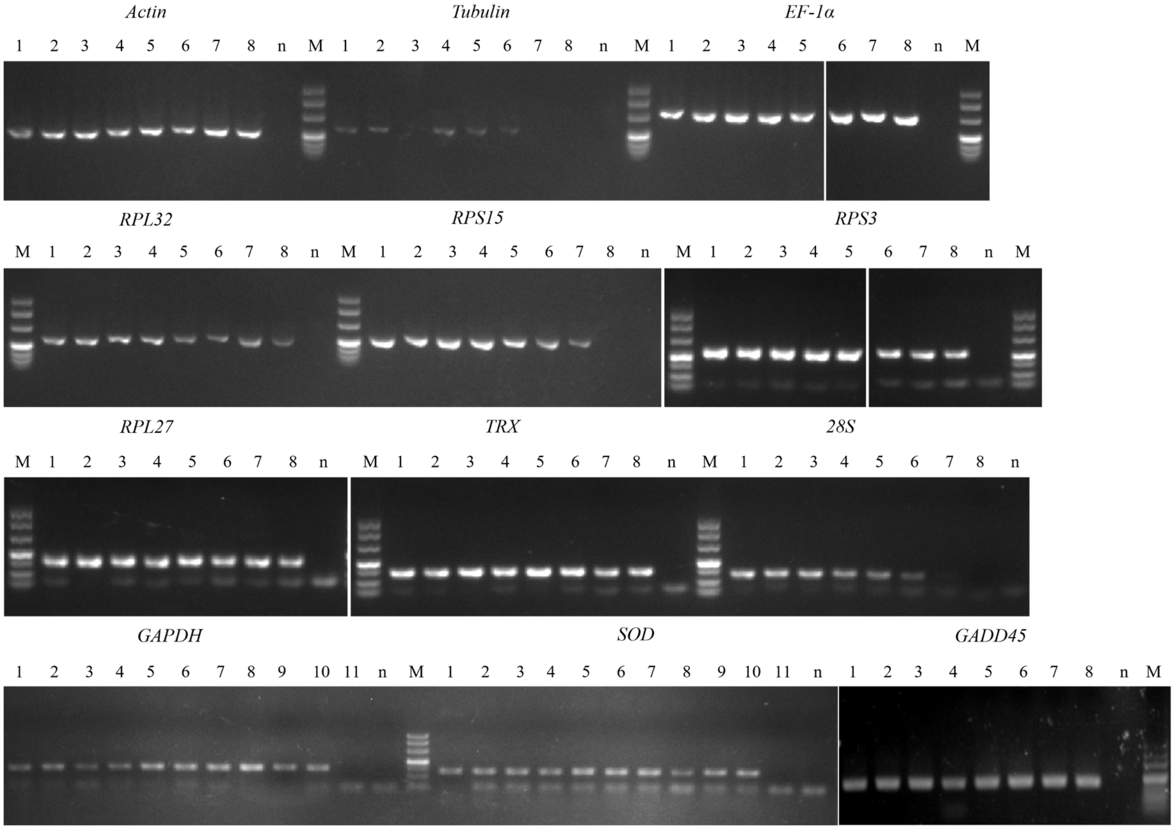

Figure S1. The annealing temperature analysis of qPCR amplification of eleven candidate genes and one target gene in *H. armigera*. Line 1-8 represent the 51.6, 53.1, 54.9, 56.6, 58.2, 60.3, 62.0 and 63.8 ℃ of *Actin*, *Tubulin*, *EF-1α*, *RPS3*, *RPS15*, *RPL27*, *RPL32*, *28S*, *TRX* and *GADD45* genes; Line 1-11 represent the 51.2, 52.8, 54.2, 55.5, 56.9, 58.0, 59.2, 60.3, 61.8, 63.0 and 63.8 ℃ of *GAPDH* and *SOD* genes; Line n represents the negative control; Line M represents DL500 DNA Marker (Takara).

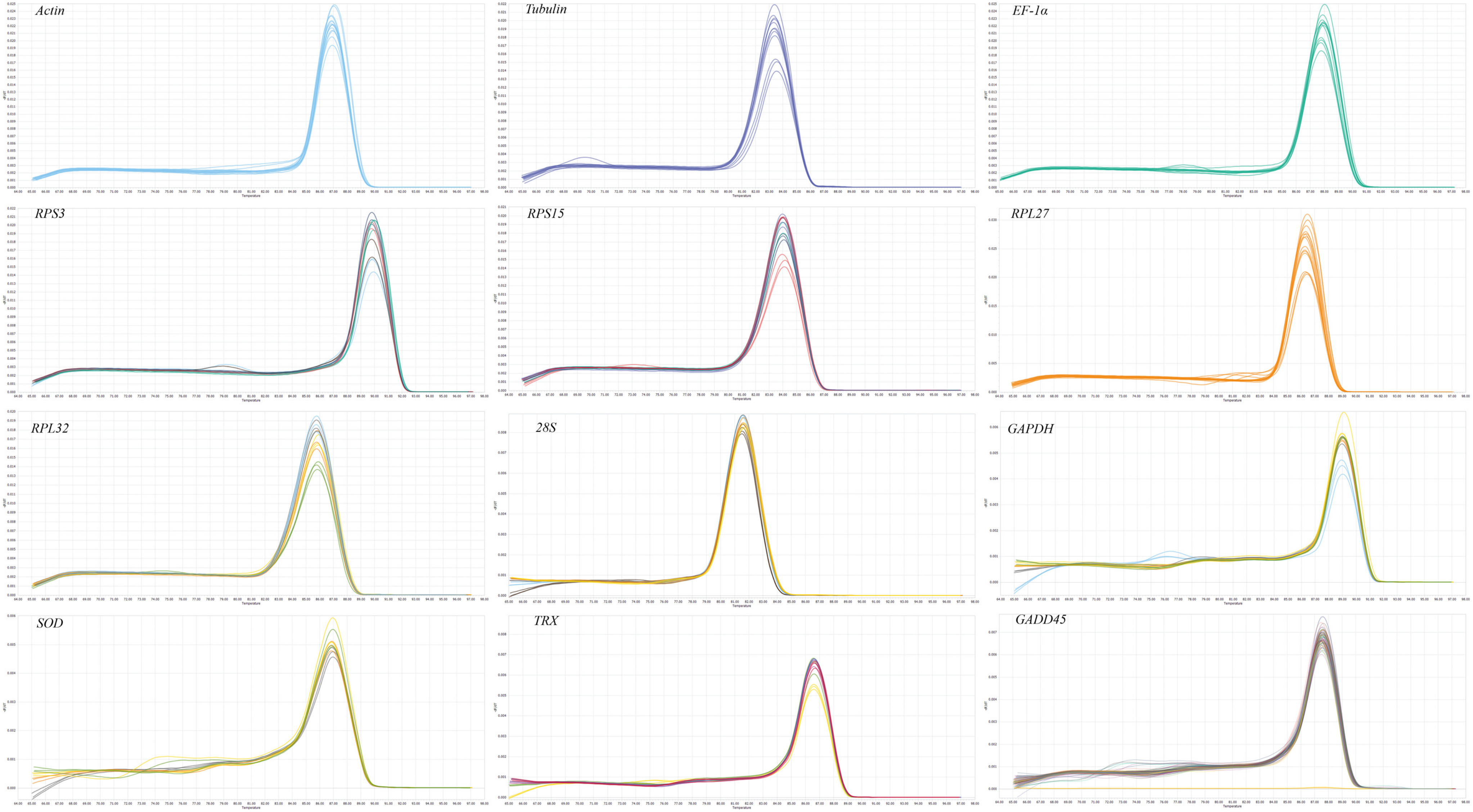

Figure S2. The melt curve analysis of qPCR amplification of eleven candidate genes and one target gene in *H. armigera*, including *Actin*, *Tubulin*, *EF-1α*, *RPS3*, *RPS15*, *RPL27*, *RPL32*, *28S*, *GAPDH*, *SOD*, *TRX* and *GADD45*.
